# Exposure to Feline Viruses in European Wildcats (*Felis s. silvestris*) in Germany: Spatial Patterns and Environmental Risk Factors

**DOI:** 10.3390/v18060627

**Published:** 2026-05-29

**Authors:** Mike Heddergott, Jutta Pikalo, Franz Müller, Peter Steinbach, Julian Wittische, Sandra Steeb, Diana Jeschke, Ole Anders, Hermann Ansorge, Alain C. Frantz

**Affiliations:** 1 Department of Zoology, Musée National d’Histoire Naturelle, 25, rue Muenster, 2160 Luxembourg, Luxembourg; psteinbach@web.de (P.S.); jwittische@gmail.com (J.W.); afrantz@mnhn.lu (A.C.F.); 2 Institute of Parasitology, University of Veterinary Medicine Vienna, Veterinärplatz 1, 1210 Vienna, Austria; 3 Working Group for Wildlife Reserach, Justus-Liebig-University Giessen, Frankfurter Straße 114, 35392 Giessen, Germanysandrasteeb@yahoo.de (S.S.); 4 Faculty of Chemistry, Georg-August University of Göttingen, Tammannstraße 4, 37073 Göttingen, Germany; 5 Senckenberg Museum of Natural History Görlitz, Am Museum 1, 02826 Görlitz, Germany; diana.jeschke@senckenberg.de (D.J.); hermann.ansorge@senckenberg.de (H.A.); 6 Nationalpark Harz, Erzwäsche 1, 37444 Sankt Andreasberg, Germany; ole.anders@npharz.de; 7 International Institute Zittau, Technische Universität Dresden, Markt 23, 02763 Zittau, Germany

**Keywords:** disease spillover, feline leukemia virus, feline parvovirus, seroprevalence, spatial epidemiology, wildlife conservation

## Abstract

While viral diseases of domestic cats (*Felis catus*) can threaten the recovery of the European wildcat (*Felis s. silvestris*), their epidemiology in wildcat populations remains poorly understood. Here, we analyzed 428 road-killed wildcats from Western and Central Germany for the presence of antibodies/antigens of six feline viruses. The presence of at least one viral antigen or antiviral antibody was detected in 53.3% of the animals. Antibodies against feline parvovirus (FPV) showed the highest seroprevalence (29.2%), while feline leukemia virus (FeLV) antigens were detected in 22.2% of the animals. Antibodies to feline coronavirus (FCoV), feline herpesvirus (FHV) and feline calicivirus (FCV) were detected in 10% or fewer of the wildcats. No antibodies to feline immunodeficiency virus (FIV) were detected. FeLV antigens clustered spatially, with prevalence declining from southwest to northeast, consistent with the geographic expansion of the virus antigens. Seroprevalence of FPV and prevalence of FeLV increased with age, suggesting cumulative exposure, while juvenile males were particularly unlikely to be seropositive for FPV. Proximity to built-up areas did not predict seroprevalence for any virus. FeLV and FPV in particular warrant further investigation as potential threats to wildcat recovery in Germany and highlight the need for longitudinal health monitoring alongside existing conservation efforts.

## 1. Introduction

Viral diseases can negatively impact the distribution and abundance of animals and, through interaction with other drivers of decline, become a significant threat to endangered taxa [1,2]. As a result of habitat destruction and anthropogenic habitat modifications, as well as the introduction of non-native species, the contact rate between domestic animals and their wild relatives has increased, which may lead to an increase in the exchange of viruses and other pathogens between the two groups [3,4]. Spillover of viruses typical of domestic cats and dogs to wild members of the Canidae and Felidae can have particularly dramatic consequences [1,5,6].

Once distributed from the Iberian Peninsula to Eastern Europe, the European wildcat (*Felis s. silvestris*) has undergone a substantial population decline and range reduction through human persecution and habitat loss [7,8]. Due to strict legal protection at the European level, the species is slowly recovering and expanding its range in Western and Central Europe [9,10]. However, its conservation status is still classified as unfavorable in many European countries [11]. The recovery of the wildcat, together with a concurrent increase in numbers of domestic cats (*Felis catus*), has led to increased contact between the two, as evidenced by hybridization rates, particularly in range-edge areas with low wildcat densities [12,13]. Increasing contact between unvaccinated domestic cats and wildcats is expected to increase the exposure of wildcats to pathogens from domestic cats [6,14].

The most important viral infections in cats include the feline leukemia virus (FeLV), the feline immunodeficiency virus (FIV), the feline coronavirus (FCoV), the feline calicivirus (FCV), the feline herpesvirus (FHV) and feline parvovirus (FPV) [15]. Feline leukemia virus (FeLV) is one of the most common causes of disease-related mortality in domestic cats. Transmission of the gammaretrovirus mainly results from close contact between animals, via saliva or biting [16,17]. Domestic cats can transmit the virus to other small felids, including the Iberian lynx (*Lynx pardinus*) and wildcats [18,19]. The presence of FeLV in endangered wild felids is of particular concern because infection can cause immunosuppression, neoplasia, and hematopoietic disorders, negatively affecting the life expectancy of infected animals [6,20].

Feline immunodeficiency virus is a lentivirus causing an immunodeficiency syndrome in domestic cats that causes a gradual loss in immune function, leading to opportunistic infections as well as tumors and blood cancer [21]. As the virus is mainly transmitted through bite wounds and sometimes during mating, the infection is more common in feral males due to their aggressive interactions with other males [22]. While most strains of FIV-like viruses are species-specific [23,24], there are recorded cases of transmission from domestic to wild felids [25,26] and reports of the presence of antibodies in some populations of the wildcat [27,28,29].

The feline coronavirus is mainly transmitted by the fecal-oral route, with infection facilitated by close contact and multi-cat environments [30,31]. Kittens are most at risk of infection, particularly when maternal antibody levels decline [32]. Most infections are subclinical or cause a mild form of enteritis. However, the virus can mutate within the host and cause feline infectious peritonitis (FIP), an often invariably fatal immune-mediated inflammatory disease [32,33]. Generally, less than 10% of FCoV-infected individuals develop FIP [34]. Feline coronavirus can be transmitted from domestic cats to wild felids [35]. The virus has mostly been detected in captive felids [36], in which it similarly can cause fatal FIP [35,37].

Feline calicivirus and feline herpesvirus 1 (FHV-1) are the most common viruses causing upper respiratory tract disease in domestic cats [38]. Both are highly contagious and transmitted through contact with oronasal and conjunctival secretions from infected animals [39,40]. Alongside sneezing and nasal discharge, FCV causes oral ulcers, and sometimes pneumonia, enteritis or transient lameness [39,41]. Feline calicivirus is a non-enveloped RNA virus that can mutate rapidly [42]. Some FCV strains are highly virulent with a high mortality rate, but the prevalence of these strains is low, with outbreaks occurring sporadically in high-density environments [41,43]. The virus has been isolated from wildcats [44] and is also known to occur in other wild felids [45,46], with evidence of transmission from domestic cats [47].

Alongside upper respiratory disease, FHV-1 can cause conjunctival and corneal ulceration and other ocular conditions in cats [48]. Infection with the virus is generally non-fatal in adult cats. However, in high-density or unvaccinated settings, outbreaks with kitten mortality can occur [40]. Feline herpesvirus 1 is known to occur in the European wildcat [19] as well as in a number of other wild felids, in which case fatalities and evidence of spillover from domestic cats have sometimes been reported [49,50,51].

Feline panleukopenia virus is a protoparvovirus that causes panleukopenia, a disease characterized by a low white blood cell count, enteritis, dehydration and secondary bacterial infections often leading to death [52,53]. Kittens, which are particularly susceptible after the decline of maternal antibody levels, have mortality rates of over 90% if left untreated [52]. Also, congenital or neonatal infections can lead to permanent cerebellar damage [54]. The virus is highly contagious, and all body secretions and excrements are infectious, while its high environmental stability allows efficient indirect transmission [53]. Feline panleukopenia virus causes a similar disease in wild felids and fatal outbreaks have been reported from captive settings [42,55,56]. Seroprevalence of FPV antibodies can reach up to 29% in European wildcats [19]. There is evidence of cross-species transmission of the virus and European wildcats, and other carnivores, can also be infected with canine strains of the virus [56,57,58].

The main threats to the continued recovery of the European wildcat in Western and Central Europe are generally considered to be habitat fragmentation, hybridization with domestic cats, and road traffic mortality [59]. Although the severity of viral infections in the European wildcat is not well understood, disease may be an additional stressor negatively impacting population viability. Exposure to FeLV and FPV can reduce body condition in wildcats, suggesting that both viruses may cause severe disease and increased mortality in this species [27,60]. While FCoV, FCV, and FHV-1 mainly cause mild or even subclinical disease in domestic cats [34,39,40], FCoV can be fatal in young captive wildcats [61] and co-infections may worsen disease severity [52,62].

Despite its potential relevance in population persistence and recovery, the epidemiology of domestic cat viruses in wildcat populations remains understudied. Therefore, the overall objective of the present study was to gain a better understanding of the dynamics or viral diseases in European wildcat populations. We generated data on the presence of FeLV antigens and antibodies against FIV, FCoV, FCV, FHV and FPV from a large number of German wildcats to investigate spatial patterns of viral transmission and risk factors driving infection. We generated maps of exposure patterns to highlight areas of elevated infection risk and tested for biological, spatial and environmental factors facilitating transmission.

## 2. Materials and Methods

### 2.1. Ethical Statement

In accordance with Directive 2010/63/EU of the European Parliament and of the Council of 22 September 2010, on the protection of animals used for scientific purposes, no ethics approval was required for this type of study.

### 2.2. Sample Collection

**Figure 1:**
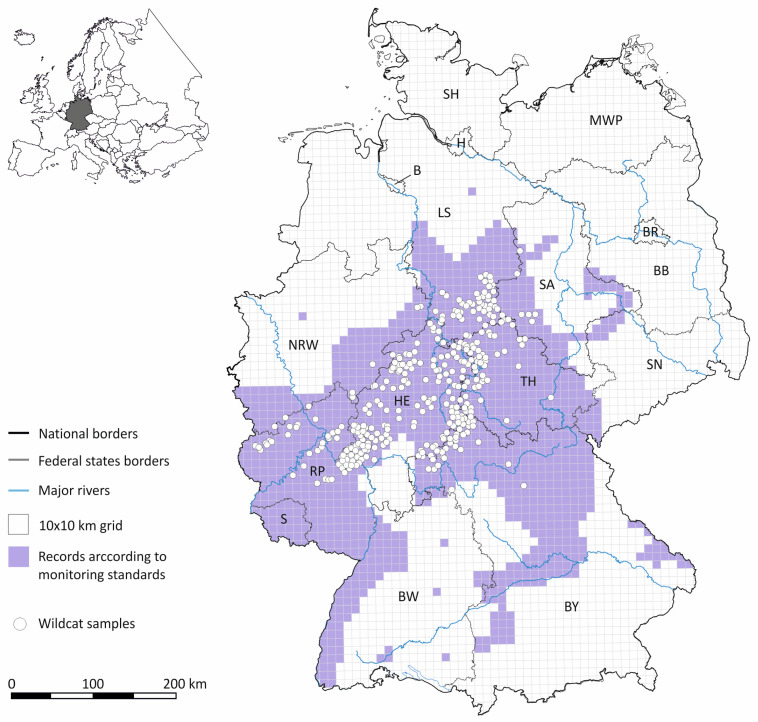
Geographic origin of the 428 European wildcats (*Felis s. silvestris*) from Germany included in this study. The blue area represents the geographic distribution of wildcats in Germany according to the National FFH Report 2019, plotted on the 10 × 10 km reference grid ETRS89-LAEA5210 EEA according to a compilation of the German Federal Agency for Nature Conservation (BfN) and monitoring data of the federal states [63]. Abbreviations: Brandenburg (BB), Bremen (B), Berlin (BR), Baden-Württemberg (BW), Bavaria (BY), Hamburg (H), Hesse (HE), Mecklenburg-West Pomerania (MWP), Lower Saxony (LS), North Rhine-Westphalia (NRW), Rhineland-Palatinate (RP), Schleswig-Holstein (SH), Saarland (S), Saxony (SN), Saxony-Anhalt (SA) and Thuringia (TH).

Between 1995 and 2024, 428 road-killed wildcats were collected in Western and Central Germany and stored at −20 °C until dissection. The collection was carried out as part of a carcass monitoring program. The organizations involved included regional hunting associations (Landesjagdverbände, LJV), the regional branches of the Bund für Umwelt und Naturschutz Deutschland (BUND; Friends of the Earth Germany), the regional branches of the Naturschutzbund Deutschland (NABU; Nature and Biodiversity Conservation Union), the Harz National Park, the ‘FELIS project’ of the Wildlife Biology Working Group at Justus Liebig University of Giessen, the Senckenberg Society of Nature History Görlitz, and the state nature conservation authorities. The animals originated from six federal states: Bavaria (*n* = 26), Hesse (*n* = 249), Lower Saxony (*n* = 53), North Rhine–Westphalia (*n* = 4), Rhineland-Palatinate (*n* = 22), Saxony-Anhalt (*n* = 4), and Thuringia (*n* = 70) (Figure 1).

During the necropsy, 1–10 mL of blood was collected from the heart or abdominal cavity of each animal [19], along with various other samples (e.g., spleen) for further analysis, and the sex of each animal was recorded. Of the individuals examined here, 116 were included in other studies on vector-borne pathogens [64,65]. Blood samples were centrifuged at 1000 *g* for 15 min using an EBA 200 benchtop centrifuge (Hettich, Tuttlingen, Germany) and sera were stored −20 °C [66,67]. The cats were classified as wildcats based on various morphological characteristics [68,69], the intestinal index [70], and the cranial index [71]. Individuals for which classification was ambiguous or not possible due to severe skull damage or advanced decomposition, as well as those with suspected hybridization with domestic cats, were analyzed genetically [9,72].

The age of most of the animals (*n* = 324) was determined based on incremental growth lines in the cementum of a canine from the lower jaw [73]. After demineralization with 5% nitric acid (HNO_3_), teeth were cross-sectioned (width, 5 μm) using a rotary microtome (RM 2050, Leica Biosystems Nussloch GmbH, Nussloch, Germany) and stained with hematoxylineosin. A B1-220A light microscope (Motic, Wetzlar, Germany) was used to count growth lines at ×40–100 magnification. Based on Piechocki and Stiefel [74], animals without growth lines were classified as juveniles (<12 months old), individuals with one growth line were considered subadults (12–24 months), whereas those ≥25 months old with two or more growth lines were classified as adults. For a subset of animals from museum collections (*n* = 104) that consisted of complete skull specimens with full dentition, age was determined by measuring the dental pulp from X-ray images of a canine tooth from the lower jaw [75], with the categorization again following Piechocki and Stiefel [74]. The dataset consisted of 263 adults, 93 subadults and 72 juveniles, as well as 275 males and 153 females.

### 2.3. Virus Detection

To detect FeLV antigen in sera, the SNAP^®^ FIV/FeLV Combo Test targeting the P27 antigen of FeLV was used according to the manufacturer’s instructions (IDEXX Laboratories Inc., Westbrook, ME, USA). According to the manufacturer, the test has a sensitivity of 98.6% and a specificity of 98.2% for FeLV antigen detection.

### 2.4. Antibody Detection

To detect FIV-specific antibodies, the SNAP^®^ FIV/FeLV Combo Test (IDEXX Laboratories Inc., Westbrook, ME, USA), which uses inactivated gag and env (gp40) antigens, was used according to the manufacturer’s instructions. According to the manufacturer, the test has a sensitivity of 93.5% and a specificity of 100% for the detection of antibodies against FIV.

Antibodies against FCoV, FCV, FHV and FPV were identified by indirect immunofluorescence tests (IFAT) using various MegaFLUO ^®^ tests (company Diagnostik Megacor, Hörbranz, Austria): FCoV (MegaFLUO^®^ FCoV; sensitivity 100%; specificity 84%), FCV (MegaFLUO^®^ FCV), FHV (MegaFLUO^®^ FHV) and FPV (MegaFLUO^®^ PAN) according to the manufacturer’s instructions. Sera with a titer of 1:20 (FPV) and 1:40 (FCV, FHV) and 1:100 (FCoV) or higher were considered positive according to the manufacturer’s instructions, and those with doubtful results were re-tested. Each procedure incorporated positive and negative control sera according to the manufacturer’s instructions.

### 2.5. Statistical Methods

All statistical analyses were performed in the program R v.4.2.2 [76]. We employed the package binom v.1.1.1.1 [77] to use the Wilson procedure with a correction for continuity to calculate the 95% confidence intervals (CI) of the proportion of seropositive animals [78]. The spatial autocorrelation of viral seroprevalence was analyzed using Moran’s I [79] in the R package spdep v.1.3.5 [80]. Given that the sampling was not uniform and since we did not have a biologically meaningful distance threshold, we generated a spatial network using *k*-nearest neighbors (*k*-nn), employing spdep v.1.3.5. We chose the eight nearest neighbors (*k* = 8) in order to capture both local and broader scale spatial patterns. We used the package ggplot2 v3.5.1 [81] to generate a map with hotspots of high antibody prevalence based on kernel density estimation (KDE), applied to positive cases using the stat_density_2d() function.

We fitted logistic regressions with generalised linear mixed effects models (GLMMs) in the glmmTMB package [82] to test for the effect of latitude, longitude, sex and age class on the probability of antigen or antibody presence for each virus, except for FIV (no antibodies detected) and FCV (very low antibody prevalence; see Results). Given the relatively large spatial scale over which our samples were collected, we also wanted to include environmental variables as fixed effects and explicitly account for spatial autocorrelation. We used 16 climatic variables (Table S1) obtained from the open data portal (https://opendata.dwd.de/ (accessed on 18 December 2024)) of the German Meteorological Service (Deutscher Wetterdienst, DWD). The data was in the form of 1 × 1 km ascii grids and clipped to each sampling point using the extract() command of the package raster v.3.6-26 [83]. We used the Shuttle Radar Topography Mission (SRTM)-based digital terrain model (DTM) map of Germany (https://www.opendem.info; (accessed on 18 December 2024)) to similarly determine the elevation of each sampling site.

A high contact rate between domestic cats and wildcats likely increases the risk of virus transmission [19]. Since the density of free-roaming and feral domestic cats is highest in proximity to human settlements [84,85], we used the distance to the nearest built-up area as a proxy for domestic cat density, basing ourselves on a 10 × 10 m 2020 landcover map generated from Sentinel-2 data (https://www.mundialis.de/en/germany-2020-land-cover-based-on-sentinel-2-data/; (accessed on 18 December 2024)). For each data point, we created a circle with a radius of 5 km, clipped the landcover map to this area, converted the resulting raster to a polygon and calculated the distance from the sampling point to the nearest built-up area using the st_distance() command of package sf v. 1.0.16 [86]. To avoid collinearity issues, we calculated Pearson’s correlation coefficient between all pairs of environmental variables. We aimed to exclude variables with *r* > 0.7 to reduce redundancy and retained core climatic variables, including precipitation and mean temperature, rather than supplementary variables like the number of frost days and summer days.

Spatial autocorrelation in epidemiological data can confound characterization of environmental or host-related effects [87]. It can be controlled for in statistical modeling by the inclusion as fixed factors of synthetic spatial variables such as Moran’s eigenvector maps (MEMs; [88,89,90,91]). Moran’s eigenvector maps analysis is a multi-scale method that generates uncorrelated spatial eigenfunctions that can be used to identify spatial patterns of variation in response variables across a range of spatial scales. The first few MEM variables produced in the analysis, characterized by large Moran’s I coefficients, capture broad-scale processes. Subsequent MEM variables capture processes at smaller spatial scales, with lower Moran’s I coefficients reflecting fine spatial autocorrelation driven by local processes.

Moran’s eigenvector maps are derived as eigenvectors from a spatial weighting matrix constructed using the geographic coordinates of the sampling sites. We used the nb2listw() command from the package spdep to generate a spatial weighting matrix from a neighborhood list taken from the *k*-nn analysis and created the MEMs using the mem() command of the package adespatial v. 0.3.24 [92]. While MEMs can represent either positive or negative spatial autocorrelation, we limited our analysis here to positive patterns. To allow meaningful analysis, we focused on the first 100 MEMs, as extremely small-scale variables can be difficult to interpret and may have limited biological relevance in the present study of a vagile carnivore. While accurate conclusions about biology cannot always be directly derived from MEMs, they can help uncover unknown but important environmental variables varying at similar scales across the landscape and therefore help uncover spatial dependence (e.g., external abiotic variables influencing the virus), or true spatial autocorrelation (e.g., patterns of seropositivity created by disease dynamics). MEMs have successfully been used in a wide range of studies in biology and are a flexible tool [93,94,95].

In order to identify MEMs that represented meaningful spatial autocorrelation patterns, we first predicted the probability of antibody presence for each virus using logistic regressions with only MEMs as fixed factors. For ease of processing, we grouped MEMs into batches of 10, based on their scale (e.g., the first ten, then the next ten and so on). We then performed a likelihood ratio test to compare the full model for each batch with the null model. If the full model did not provide a better fit, no MEM from that batch was carried forward to the main analysis. If the full model performed better, we then used the dredge() function of the package MuMIn v.1.48.4 [96] to estimate AICc values for all possible models and selected those models whose AICc values were within 2 AICc units of the model with the lowest AICc. We carried forward to the main analysis the MEMs that were statistically significant in the most parsimonious model (lowest degrees of freedom) from this subset.

We estimated the variation inflation factors (VIFs) with the full GLMMs (including coordinates, host-related and environmental variables, as well as MEMs) without interactions to test for multicollinearity. We considered that the independent variables exhibited no significant collinearity when VIF values were <5 [97]. To avoid convergence and fitting issues, and to facilitate interpretation, we included only the interaction between *sex* and *age class* in the full model. If the full model with the interaction did not converge, we repeated the analysis with a reduced model containing only the significant factors from the initial analysis plus the interaction. We considered the year of sampling as a random effect (random intercept) to account for inter-annual stochasticity. We evaluated whether inclusion of this random effect improved the model by comparing the Akaike information criterion (AIC) of models with and without the random effect. As described above, we used the dredge() function to estimate AICc values for all possible models and selected the most parsimonious one from the subset of models that were within 2 AICc units of the model with the lowest AICc. To confirm the importance of the predictors in the most parsimonious model, we also conducted model averaging using the model.avg() function of the MuMIn package. However, we performed this model averaging only on models without interactions [98]. We plotted the marginal effects of the most parsimonious models using the plot_model() function of the sjPlot v.2.8.10 R package [99]. To assess potential overfitting and model generalizability, we evaluated the predictive performance of the final GLMs using repeated 5-fold cross-validation (100 repetitions). For each iteration, models were fitted on training subsets and evaluated on held-out data using the area under the receiver operating characteristic curve (AUC).

## 3. Results

Viral antibodies or antigens for at least one virus were found in 228/428 wildcats (53.3%; 95% CI: 48.5–57.9). Feline leukemia virus antigen was detected in 95/428 wildcats (22.2%; 95% CI: 18.5–26.4). Antibodies against FPV were found in 125 (29.2%; 95% CI: 25.1–33.9), against FCoV in 39 (9.1%; 95% CI: 6.7–12.2), against FHV in 43 (10.0%; 95% CI: 7.5–13.3) and against FCV in 10 (2.3%; 95% CI: 1.3–4.2) of the 428 tested wildcats. Antibodies against FIV were not detected in any wildcat. Antibodies against only one virus were found in 157/428 (36.7%; 95% CI: 32.3–41.3) wildcats and antibodies against two viruses in 60/428 (14.0%; 95% CI: 11.0–17.6) animals (Table 1). Nine out of 428 wildcats (2.1%; 95% CI: 1.1–3.9) were infected with FeLV and had antibodies against two other viruses, while 2/428 (0.5%; 95% CI: 0.1–1.9) wildcats were infected with FeLV and had antibodies against three other pathogens (FPV, FHV, FCV; Table 1).

**Figure 2:**
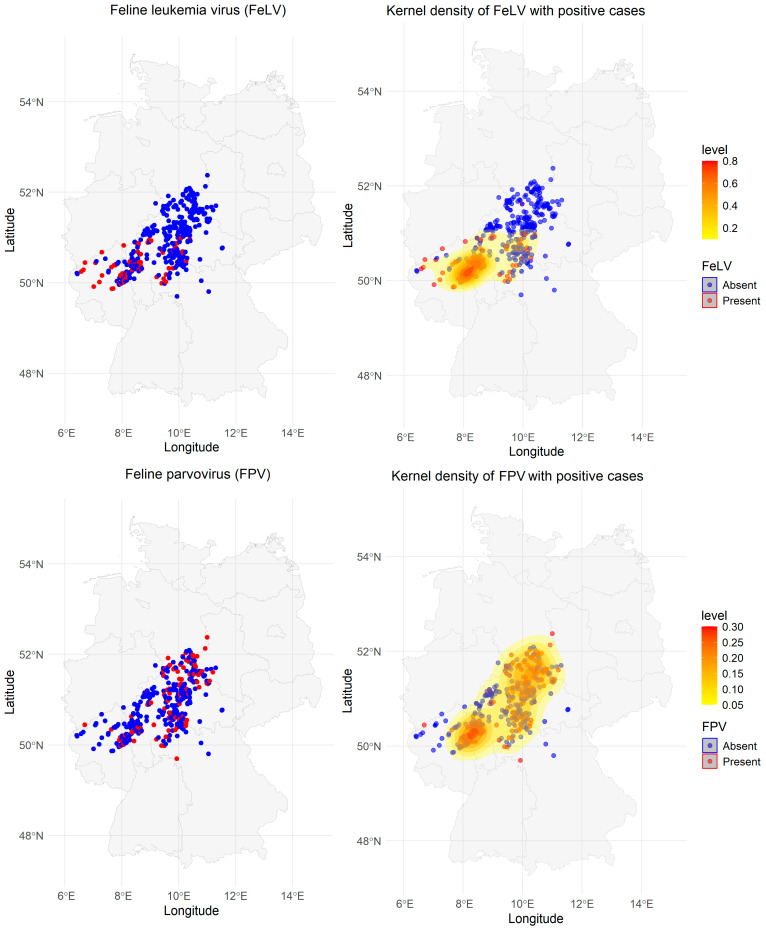
Geographic distribution and hotspots of feline leukemia virus (FeLV) antigen and antibodies against feline parvovirus (FPV) in 428 European wildcats (*Felis s. silvestris*) in Germany. The presence of antigen or antibodies was detected through serological testing. Hotspots with high densities were detected using kernel density estimation. The scale of the heatmap indicates the prevalence of individuals with antigen/antibodies. The borders of the German federal states are shown for geographic reference.

**Figure 3:**
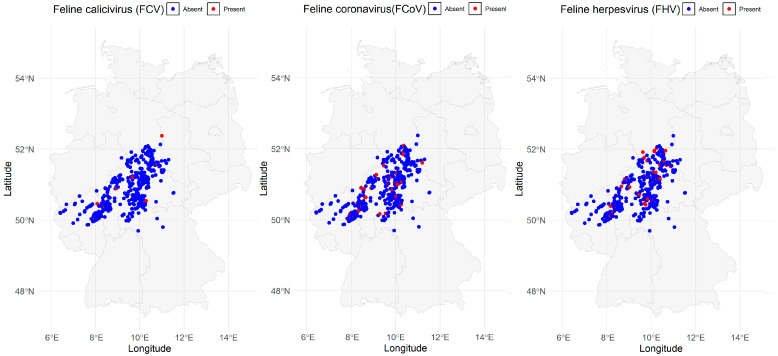
Geographic distribution of antibodies to feline calicivirus (FCV), feline coronavirus (FCoV) and feline herpesvirus (FHV) in 428 European wildcats (*Felis s. silvestris*) in Germany. The presence of the antibodies was detected through serological testing. The borders of the German federal states are shown for geographic reference.

We observed a significant geographic clustering of FeLV antigen (Moran’s I = 0.241, *p* < 0.001), with the prevalence of the antigens particularly high in the southwest of the study area (Figure 2). Antibodies for FPV exhibited a weaker, yet significant geographic clustering (Moran’s I = 0.051, *p* = 0.008), with a higher prevalence of the antibodies in the southwest and northeast of the study area (Figure 2). Antibodies to FCoV (Moran’s I = 0.010, *p* = 0.283), FHV (Moran’s I = 0.003, *p* = 0.410) and FCV (Moran’s I = 0.015, *p* = 0.211) exhibited no significant geographic clustering (Figure 3).

After excluding highly correlated climatic variables (*r* > 0.7), we retained *mean air temperature*, *precipitation*, *sunshine duration*, *elevation* and *distance to built-up areas*. In a GLMM predicting FeLV antigen presence/absence, elevation (VIF: 8.05) and mean air temperature (VIF: 11.75) showed high collinearity. Removal of *elevation* reduced all VIF values to <5, leaving four environmental variables, as well as *latitude*, *longitude*, *sex* and *age class* as fixed factors for further analyses. Moreover, 114 positive spatial eigenvectors, represented as Moran’s Eigenvector Maps (MEMs), were identified. We considered the first 100 of these in our analyses.

**Figure 4:**
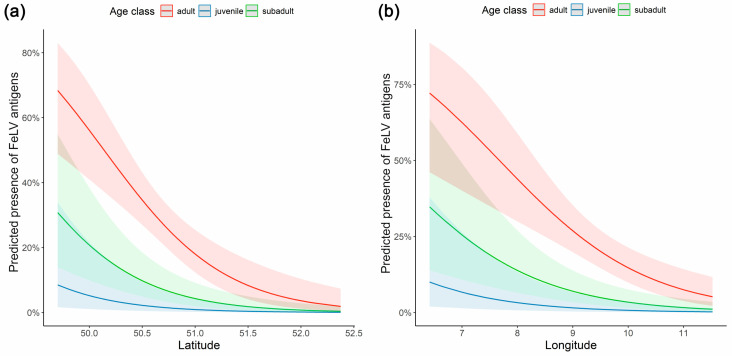
Marginal effects plot of a logistic regression model predicting the presence of feline leukemia virus (FeLV) antigens in European wildcats (*Felis s. silvestris*) as a function of (**a**) age class of the host and latitude and (**b**) age class of the host and longitude. The 95% confidence intervals are shown in color and the plots are based on the most parsimonious model identified after model selection (see Table 2).

Using GLMMs to identify spatial eigenvectors predicting the presence of antigen to FeLV, we retained ten MEMs (all representing broader-scale processes; Figure S1), leaving us with 18 fixed factors for inclusion in the full FeLV model. Due to convergence problems, we did not initially consider an interaction between *sex* and *age class*. Except for latitude (VIF: 5.84), all VIF values were <5, so all factors were retained in the model. Inclusion of *year* as a random factor did not improve the model. Our model selection procedure resulted in 25 equivalent models based on AICc. According to the most parsimonious model, adults were more likely to have FeLV antigen than juveniles or subadults. Moreover, the probability of having FeLV antigen increased with decreasing latitude and longitude, i.e., from north to south and from east to west (Table 2, Figure 4).

Model averaging confirmed the importance of all three predictors (Table S2). The marginal *R*^2^ of 0.355 indicated that the most parsimonious model had a good ability to explain the presence of FeLV antigen in German wildcats based on our fixed effects predictors. To test for a possible interaction between *sex* and *age class*, we repeated the analysis, but excluded all other fixed factors, except *latitude* and *longitude*, from the model. Our model selection procedure resulted in three equivalent models based on AICc, but the most parsimonious model was the same as the one obtained with the selection procedure starting with the full model without interactions (Table 2).

**Figure 5:**
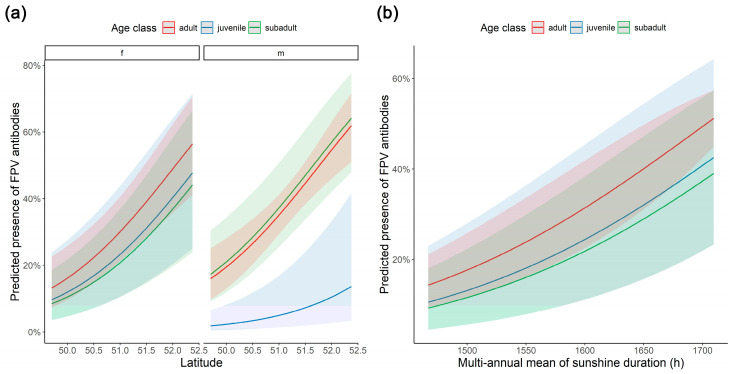
Marginal effects plot of a logistic regression model predicting the presence of feline parvovirus (FPV) antibodies in European wildcats (*Felis s. silvestris*) as a function of (**a**) age class and sex of the host, as well as latitude and (**b**) the multi-annual mean of sunshine duration and age class. The 95% confidence intervals are shown in color and the plot is based on the most parsimonious model identified after model selection (see Table 3).

Using GLMMs to identify spatial eigenvectors predicting the presence of antibodies to FPV, we retained a single MEM 10 (representing a broader-scale process; Figure S2), leaving us with nine fixed factors for inclusion in the full FPV antibody model plus the interaction between *sex* and *age class* (Table 3, Figure 5). All VIF values were <5 in a full.

GLMM without interactions. Inclusion of *year* as a random factor did not improve the model. Our model selection procedure resulted in six equivalent models based on AICc. According to the most parsimonious model, the probability of having FPV antibodies increased with latitude, decreased with increasing values of the spatial Eigenvector (MEM10) and increased with increasing duration of sunshine. There was also a significant interaction between sex and age class, with juvenile males being less likely to be seropositive than other combinations of *sex* and *age class* (Table 3, Figure 5). Model averaging performed after a model selection procedure on a model without interactions confirmed the importance of *latitude*, *MEM10* and *sunshine duration* as predictors, as well as of *age class* (Table S3). The marginal *R*^2^ of 0.123 indicates that the most parsimonious model had moderate power to predict accurately the presence of FPV antibodies in German wildcats based on our fixed effects predictors.

Using GLMMs to identify spatial eigenvectors predicting the presence of antibodies to FCoV, we retained a six MEMs (two representing a broad-scale and four finer-scale processes; Figure S3), leaving us with 14 fixed factors for inclusion in the full FCoV model. Due to convergence problems, we did not consider an interaction between sex and age class. All VIF values were <5 in a full GLMM without interactions. Inclusion of year as random factor did not improve the model. Our model selection procedure resulted in nine equivalent models based on AICc. According to the most parsimonious model, adults were more likely to have antibodies to FCoV than juveniles, the probability of having FCoV antibodies decreased with increasing values of the spatial Eigenvectors MEM17, MEM72, MEM74, MEM78, and increased with increasing values of the spatial Eigenvector MEM75 (Table 4, Figure S4). Model averaging confirmed the importance of all six predictors (Table S4). The marginal *R*^2^ of 0.122 indicates that the most parsimonious model had moderate power to predict accurately the presence of FCoV antibodies in German wildcats based on our fixed effects predictors. To test for a possible interaction between sex and age class, we repeated the analysis, but excluded all non-significant fixed factors except MEM17, MEM72, MEM74, MEM78 and MEM75 from the model. Our model selection procedure resulted in three equivalent models based on AICc. The most parsimonious model was again equivalent to the one obtained with the selection procedure based on the full model without interactions (Table 4).

Using GLMMs to identify spatial eigenvectors predicting the presence of antibodies to FHV, we retained two MEMs (representing a fine-scale process; Figure S5), leaving us with ten fixed factors for inclusion in the full FHV antibody model, plus the interaction between *sex* and *age class*. All VIF values were <5 in a full GLMM without interactions. Inclusion of *year* as a random factor caused convergence problems, and we thus omitted the random factor. Our model selection procedure resulted in 20 equivalent models based on AICc. The marginal *R*^2^ of 0.022 indicates that the most parsimonious model had a very limited power to predict accurately the presence of FPV antibodies in German wildcats based on our fixed effects predictors (Table 5).

Cross-validated predictive performance was moderate to high and consistent across folds, and close to that of the full dataset prediction, with mean AUC values of 0.87 ± 0.005 for FeLV antigens, 0.69 ± 0.011 for FPV antibody, and 0.75 ± 0.005 for FCoV antibody, indicating no substantial overfitting in our analyses.

## 4. Discussion

Here, we confirm the presence of antibodies against several feline viruses and viral antigens in a significant number of European wildcats sampled over a larger spatial scale in Western and Central Germany. Around half of the analyzed 428 cats had antigens or antibodies to at least one of the tested feline viruses.

It should be noted that the antibody and antigen test systems used in this study were originally developed and validated for domestic cats. Nevertheless, a previous study has indicated that they are also applicable to wild felids [19]. However, domestic cats and wildcats are closely related and share the same viruses [1,5,6,18,19]. It can therefore be assumed that the impact on the test systems is minimal. However, the possibility of underestimating seropositivity due to the IFAT cut-off values in some cases cannot be excluded.

Based on the presence of FPV antibodies and FeLV antigens, respectively, FPV and FeLV were the two most prevalent viral pathogens in our study area. Alongside the value reported from Luxembourg wildcats (29.4%), the prevalence of FPV antibodies in the present study (29.2%) is the highest reported in the literature [44,60,100]. Stubbe et al. [101] reported a seroprevalence of 13.7% in Rhineland-Palatinate, the German federal state that also forms the southwestern limit of our study area. Our results indicate that there is a hotspot of FPV antibody presence along the border between Rhineland-Palatinate and its neighboring state Hesse. Because this hotspot is spatially restricted, it is possibly underrepresented in state-wide estimates, which may explain the lower overall seroprevalence reported by Stubbe et al. [101]. Prevalence values of FeLV antigens reported from other European wildcat populations tend to be higher, sometimes substantially so, but these estimates were often based on much smaller sample sizes [19,27,51,60,100,101]. Also, there is evidence of a decline in antigen prevalence from the south to the north in Western/Central Europe (see below).

Antibodies to FCoV and FHV were detected in about 10% of the sampled wildcats. Ignoring one study with high prevalence values for both viruses but low sample size (*n* = 5; [51]), the seroprevalence values observed here are within the range of values observed in other studies (FCoV-Ab: 0–47.1%; FHV-Ab: 4.0–27.3%; [19,29,44,60,100,101]). We detected antibodies to FCV in only 2.3% of the analyzed wildcats, which is much lower than the seroprevalence values observed in other studies [29,44,60,100]. Stubbe et al. [101] detected FCV antibodies in 20.6% of wildcats sampled in Rhineland-Palatinate, which also forms the southwestern limit of our study area. Since Heddergott et al. [19] reported a prevalence of FCV antibodies of 29.4% from Luxembourg, which borders Rhineland-Palatinate in the west, it appears that there may also be a decline in FCV-Ab prevalence from Luxembourg to the northeast of our study area. Finally, in line with almost all other studies, we did not detect any antibodies to FIV in our wildcat sera [19,27,44,60,100,101]. The proportion of the wildcats having antibodies/antigens for more than one virus (16.6%) is lower than the value observed in other studies, where the antigen and antibody prevalence of the individual viruses tended to be higher as well [19,60,101].

The data for FeLV-Ag, FCoV-Ab and FPV-Ab gave rise to models with a sufficiently high marginal *R*^2^ (m*R*^2^ ≥ 0.122) to be considered meaningful. There were too few positive cases of FCV antibodies to permit a valid analysis. In the case of the three interpretable models, host age had an impact on the probability of being seropositive or, in the case of FeLV, on the presence of viral antigens. In the case of the three interpretable models, host age had an impact on the probability of being seropositive. In the case of FeLV-Ag and FCoV-Ab, this probability increased with age. Heddergott et al. [19] observed a similar effect for FCoV-Ab in Luxembourg wildcats, though not for FeLV-Ag, likely because the small sample size of *n* = 34 affected the detectability of an age class effect. Stubbe et al. [101] did not observe an effect of age on the prevalence of FeLV-Ag or FCoV-Ab, which the authors attributed to a lack of statistical power. Both viruses can infect animals of all age groups. Our results suggest that the cumulative exposure increases the probability of being seropositive for both viruses. Feline coronavirus can be fatal in young captive wildcats [61], as presumably can be FeLV [102]. In a study on domestic cats, the mean age of cats that died of problems relating to FCoV infection was significantly lower than that of all other cats (e.g., [30]). Thus, it is also possible that the relative rarity of antibodies in younger animals resulted from higher virus-related mortality rates in these age groups.

In the case of antibodies against FPV, and unlike in other studies [19,101], male juveniles were particularly unlikely to be seropositive compared to other combinations of sex and age class. Male juveniles are possibly particularly susceptible to the FPV-related mortality. It is likely that, like other felines [103,104,105,106,107], natal dispersal is male-biased and young wildcat males may disperse at an earlier age than females. They are thus more likely to encounter the virus, in addition to being physiologically weaker than other age classes, with reduced immunocompetence, and thus more susceptible to the effects of the virus [108]. Alternatively, the early dispersal behavior of juvenile males may cause them to be less likely to be seropositive, as they have limited contact with conspecifics [109]. This interaction between sex and age class may only be apparent in FPV, given that the virus is highly contagious and exhibits strong environmental persistence and resistance [53]. This is not the case for FeLV [110] and FHV [48].

In addition to age class, the major factors influencing the prevalence of FeLV antigens were latitude and longitude, with no other environmental variable being significant in the GLMM. The coordinates may have been confounded with an unmeasured environmental gradient. While it is also possible that contact rates between unvaccinated domestic/feral cats and wildcats differed between different parts of our study region, it is not immediately obvious why there would be a geographic gradient in this pattern. Alternatively, the spatial variation may be the legacy of the geographic expansion of the virus. Seroprevalences suggest that the virus may have spread north-eastward from eastern central France, with antigen values ranging from 76.5% (*n* = 34; 95% CI: 60.0–87.6) in the French Jura [100] to 52.9% (*n* = 34; 95% CI: 36.7–68.5) in Luxembourg [19], 28.4% (*n* = 102; 95% CI: 19.6–37.2) in Rhineland-Palatinate [101] and 22.2% (*n* = 428; 95% CI: 18.5–26.4) for Germany in the present study. Wildcats in France, Luxembourg and Western Germany are part of the same genetic population, while the cats in Central Germany form a distinct genetic cluster [111]. Nevertheless, some Central German cats were positive for FeLV-Ag, showing that there has been some viral exchange between the two genetic populations or local spillover from domestic cats.

It remains unclear as to whether FeLV infection in wildcats requires contact with domestic cats or whether infections can be self-sustained within wildcat populations. Based on epidemiological modeling, persistence of FeLV infection requires large and stable cat populations [112], suggesting that low-density wildcat populations cannot maintain the virus without spillover from domestic cats. Duarte et al. [113] found a higher prevalence of FeLV antigens in feral cats than wildcats, concluding that contact with domestic cats led to exposure of wildcats to the pathogen. Stubbe et al. [101] observed the opposite pattern and took this as evidence for self-sustained FeLV infection in wildcats, especially since the prevalence of FeLV in German domestic cats had declined in previous years due to vaccination. However, declining prevalence in domestic cats may lead to a response in wildcat populations only after a temporal delay [114] and higher mortality of infected domestic kittens due to vertical or early-life transmission may lead to underestimation of true infection prevalence in domestic cats [115,116]. Under our interpretation of geographic expansion of the virus, our findings are also consistent with the possibility that FeLV infection in wildcats may be self-sustained; however, this cannot be distinguished with certainty from ongoing spillover without further evidence on transmission dynamics and, as suggested by Fromont et al. [27], isolation of viral strains and comparative sequencing.

In the case of FPV and FCoV, a broader-scale spatial eigenvector significantly explained part of the variability in seroprevalence, while four additional local-scale eigenvectors did so for the FCoV. The broader-scale spatial eigenvectors could represent environmental gradients that were not included in the analysis, such as habitat suitability for the host and fluctuations in host population density [117,118,119]. They could also reflect some unmeasured climatic variables, even though the ones that were tested in our analyses appeared to have relatively little impact on seroprevalence. It is possible that this result is a proxy for the time of year, as juveniles progressively lose maternally derived antibodies through spring and summer, increasing their susceptibility to infection [120]. The finer-scale spatial eigenvectors explaining variability in FCoV seroprevalence likely represent local changes in contact rates due to, for example, family links, local changes in resource distribution [121] and the location of suitable habitat.

It is striking that proximity to built-up areas was not a significant factor explaining the prevalence of the different antibodies/antigens. Taken at face value, this result could be interpreted to mean that all the viral antigens and antibodies for which statistical analysis was performed (FeLV, FPV, FCoV) were self-sustained in wildcat populations. While this may be the case for some viruses (see above), it is also possible that proximity to built-up areas was not a good proxy for feral and/or domestic cat density. Alternatively, the abundance of domestic cats alone may not be the main driver of viral transmission. Due to behavioural exclusion, differences in habitat selection and space use, encounters between domestic and wildcats might be rarer than suggested by estimates of domestic cat abundance and spatial proximity [122,123]. Finally, not all viruses require direct contact between animals for transmission. Feline Parvovirus, for instance, is shed via feces and can remain infectious in the environment for months, allowing efficient indirect transmission [53], likely making spatial proximity to built-up areas a poor predictor of seroprevalence. In order to determine whether the viruses are self-sustained in wildcat populations, phylogenetic and molecular approaches are ultimately required [26,35,50].

Among the viral antigens and antibodies analyzed in this study, FeLV-Ag and FPV-Ab appear to be of particular concern. They have a high prevalence and seroprevalence in our study area and cause clinical symptoms in wild felids like those observed in domestic cats, with documented cases of mortality [6,102], although, in the case of FPV-Ab, mainly in a captive setting [42,55,56]. In the study by Fromont et al. [27], FeLV-positive cats had a poorer body condition than the FeLV-negative cats, suggesting that FeLV may also be pathogenic in wildcats. In the case of FPV, there are three reported cases of FPV causing mortality in captive wildcats [124,125] and one study linking FPV seropositivity with reduced body condition in free-ranging wildcats [60]. In addition to these viruses, FCoV can be fatal in young captive wildcats [61], and coinfection with FCoV may exacerbate the clinical severity of FPV infection [52], while FCV and FHV-1 mainly cause mild or even subclinical disease in domestic cats [34,39,40] and, presumably, in wildcats as well. Mortality cases attributable to the investigated viruses remain undetected in free-ranging wildcats. Even though FeLV infection may be of interest as a co-infection, reducing the immunologic response in three wildcats that finally died of bacterial infection [126]. Serological surveys cannot detect active viremia, persistent infection, or any information on unsampled groups like kittens, where mortality risk is highest for some viruses [127,128].

Routine genetic monitoring of wildcats is based on hair-trapping on valerian-treated lure sticks [129]. Wildcats mark conspicuous objects with urine [130] and feces [131], and it has been shown that cats spray-mark the lure sticks [132]. Additionally, felids use secretions of different glands from the face and tail areas by rubbing to scent marks or scratching on tree bark to produce visual marks [133,134,135]. Given that FPV is highly resistant in the environment, this marking behavior could plausibly lead to FPV contamination of lure sticks and indirect transmission of the virus between cats. Given that FPV may be carried by fomites (shoes, clothing [52]), researchers may transport the virus between sites. In areas where FPV is widespread, transmission risks should be minimized by using single-use lure sticks, disinfecting reusable sticks, or relocating lure sites within a field site.

Despite the lack of information on fatalities related to the tested viruses, infectious diseases, in combination with other threats such as habitat loss, road mortality and hybridization, may have important conservation implications for recovering wildlife populations. Recovery alone does not necessarily make populations resilient to additional mortality [59,136]. Re-establishment can be threatened even by moderate parasite-induced mortality, as persistent or chronic infections may reduce body condition, survival, and reproduction and thereby constrain population growth [137,138]. Recruitment, and hence population growth, may be further limited if juveniles are particularly susceptible and mortality disproportionately affects this age class [139,140], as is the case with FCoV [32], FHV [40] and particularly FPV, where kittens have mortality rates of over 90% if left untreated [52]. High prevalence of feline viral diseases may also negatively affect population recovery of the European lynx (*Lynx lynx*), as they are known to prey both on feral domestic cats [141] and European wildcats [142] and are susceptible to spillover of feline viruses [6].

There is a lack of evidence linking the investigated viral antigens and antibodies to population-level declines in wildcats, highlighting the need for longitudinal or demographic studies that include individual life-history data, molecular diagnostics, health assessments and long-term population monitoring, rather than serological surveys or isolated case reports [143]. However, these kinds of studies are difficult to fund, set up and maintain, as they are a logistical challenge, resource-intensive and require long-term commitment. In the meantime, viral infections should be regarded as a limiting factor in the recovery of wildcat populations, and their potential impacts should be considered when analyzing population trends and designing conservation strategies.

## Figures and Tables

**Table 1:** Number of European wildcats (*Felis s. silvestris*) tested positive for antigen and antibodies against multiple viruses.

Combinations of the Antigen and Antibody Detection	Number of Wildcats
**Wildcats antigen or antibody positive for two viral diseases ^1^**	**60**
FeLV-Ag + FPV-Ab	24
FeLV-Ag + FCoV-Ab	6
FeLV-Ag + FHV-Ab	2
FeLV-Ag + FCV-Ab	1
FPV-Ab + FCoV-Ab	9
FPV-Ab + FHV-Ab	14
FPV-Ab + FCV-Ab	3
FCoV-Ab + FHV-Ab	1
**Wildcats antigen or antibody positive for three viral diseases**	**9**
FeLV-Ag + FPV-Ab + FCoV-Ab	5
FeLV-Ag + FPV-Ab + FHV-Ab	3
FeLV-Ag + FCoV-Ab + FHV-Ab	1
**Wildcats antigen or antibody positive for four viral diseases**	**2**
FeLV-Ag + FPV-Ab + FHV-Ab + FCV-Ab	2
**Total**	**71**

^1^ Ag = antigen; Ab = antibody; FeLV = feline leukemia virus; FCV = feline calicivirus; FHV = feline herpesvirus; FCoV = feline coronavirus; FPV = feline parvovirus.

**Table 2:** Logistic regression identifying predictors for the presence of antigens to feline leukemia virus (FeLV) in European wildcats (*Felis s*. *silvestris*) in Germany. Results are presented for the most parsimonious model identified after model selection.

Fixed Effects	Estimate	s.e.	*z* Value	*p* Value
(Intercept)	95.312	19.522	4.882	<0.001
Age class-juvenile	−3.144	0.778	−4.042	<0.001
Age class-subadult	−1.583	0.393	−4.031	<0.001
Latitude	−1.760	0.401	−4.386	<0.001
Longitude	−0.755	0.184	−4.114	<0.001

In the initial model, we included *latitude*, *longitude*, *sex*, *age class*, *mean air temperature*, *precipitation*, *distance to built-up areas*, as well as ten Moran’s eigenvector maps (MEMs) as fixed factors. No interactions were included due to convergence problems. In the case of the continuous predictor variables, the logistic regression coefficient gives the change in the log odds of antibody presence for a one-unit increase. In the case of the categorical variable (sex, age class), the logistic regression coefficient gives the change in the log odds of prevalence when considering males as well as juveniles and subadults relative to females and adults, respectively.

**Table 3:** Logistic regression identifying predictors for the presence of antibodies to feline parvovirus (FPV) in European wildcats (*Felis s. silvestris*) in Germany. Results are presented for the most parsimonious model identified after model selection.

Fixed Effects	Estimate	s.e.	*z* Value	*p* Value
(Intercept)	−53.674	16.565	−3.240	0.001
Age class-juvenile	−0.349	0.543	−0.642	0.521
Age class-subadult	−0.495	0.479	−1.033	0.302
Latitude	0.802	0.251	3.193	0.001
MEM10	−0.647	0.152	−4.249	<0.001
Sex-male	0.228	0.295	0.773	0.440
Sunshine duration	0.008	0.003	2.345	0.019
Age class-juvenile*Sex-male	−1.984	0.924	−2.147	0.032
Age class-subadult*Sex-male	0.590	0.590	1.001	0.317

In the initial model, we included *latitude*, *longitude*, *sex*, *age class*, *mean air temperature*, *precipitation*, *distance to built-up areas* as well as one Moran’s eigenvector map (MEM10) as fixed factors, as well as an interaction between sex and age class. In the case of the continuous predictor variables, the logistic regression coefficient gives the change in the log odds of antibody presence for a one-unit increase. In the case of the categorical variable (sex, age class), the logistic regression coefficient gives the change in the log odds of prevalence when considering males as well as juveniles and subadults relative to females and adults, respectively.

**Table 4:** Logistic regression identifying predictors for the presence of antibodies to feline coronavirus (FCoV) in European wildcats (*Felis s. silvestris*) in Germany. Results are presented for the most parsimonious model identified after model selection.

Fixed Effects	Estimate	s.e.	*z* Value	*p* Value
(Intercept)	−2.349	0.237	−9.899	<0.001
Age class-juvenile	−2.355	1.039	−2.267	0.023
Age class-subadult	−0.877	0.524	−1.674	0.094
MEM17	−0.421	0.162	−2.603	0.009
MEM72	−0.450	0.197	−2.280	0.023
MEM74	−0.498	0.184	−2.702	0.007
MEM75	0.583	0.178	3.283	0.001
MEM78	−0.406	0.177	−2.301	0.021

In the initial model, we included *latitude*, *longitude*, *sex*, *age class*, *mean air temperature*, *precipitation*, *distance to built-up areas*, as well as six Moran’s eigenvector maps (MEMs) as fixed factors. No interactions were included due to convergence problems. In the case of the continuous predictor variables, the logistic regression coefficient gives the change in the log odds of antibody presence for a one-unit increase. In the case of the categorical variable (sex, age class), the logistic regression coefficient gives the change in the log odds of prevalence when considering males as well as juveniles and subadults relative to females and adults, respectively.

**Table 5:** Logistic regression identifying predictors for the presence of antibodies to feline herpesvirus (FHV) in European wildcats (*Felis s. silvestris*) in Germany. Results are presented for the most parsimonious model identified after model selection.

Fixed Effects	Estimate	s.e.	*z* Value	*p* Value
(Intercept)	−2.284	0.173	−13.193	<0.001
MEM74	−0.335	0.170	−1.972	0.049
MEM77	0.380	0.160	2.372	0.018

In the initial model, we included *latitude*, *longitude*, *sex*, *age class*, *mean air temperature*, *precipitation*, *distance to built-up areas*, as well as two Moran’s eigenvector maps (MEMs) as fixed factors, as well as an interaction between sex and age class. In the case of the continuous predictor variables, the logistic regression coefficient gives the change in the log odds of antibody presence for a one-unit increase. In the case of the categorical variable (sex, age class), the logistic regression coefficient gives the change in the log odds of prevalence when considering males as well as juveniles and subadults relative to females and adults, respectively.

## Data Availability

The data and code used in this study are available on request from the corresponding authors.

## Supplementary Materials

The following supporting information can be downloaded at: https://www.mdpi.com/article/10.3390/v18060627/s1, Figure S1: Significant Moran’s eigenvector maps (MEMs) explaining spatial autocorrelation in feline leukemia virus (FeLV) antibody presence, as identified by a generalized linear mixed model (GLMM) with MEMs as fixed effects. Figure S2: Moran’s eigenvector map 10 (MEM10) significantly explains spatial autocorrelation in feline parvovirus (FPV) antibody presence, as identified by a generalized linear mixed model (GLMM) including MEMs as fixed effects. Figure S3: Significant Moran’s eigenvector maps (MEMs) explaining spatial autocorrelation in feline coronavirus (FCoV) antibody presence, as identified by a generalized linear mixed model (GLMM) with MEMs as fixed effects. Figure S4: Marginal effects plot of a logistic regression model predicting the presence of feline coronavirus (FCoV) antibodies in German wildcats as a function of (a) age class of the host and the spatial Eigenvector MEM75. Figure S5: Significant Moran’s eigenvector maps (MEMs) explaining spatial autocorrelation in feline herpesvirus (FHV) antibody presence, as identified by a generalized linear mixed model (GLMM) with MEMs as fixed effects. Table S1: Climatic variables used in our analysis. Table S2: Logistic regressions identifying predictors for the presence of feline leukemia virus (FeLV) antibodies in German wildcats. Table S3: Logistic regressions identifying predictors for the presence of feline parvovirus (FPV) antibodies in German wildcats. Table S4: Logistic regressions identifying predictors for the presence of feline coronavirus (FCoV) antibodies in German wildcats.

## Abbreviations

The following abbreviations are used in this manuscript:

Ab	Antibody
Ag	Antigen
AIC	Akaike information criterion
AICc	Akaike Information Criterion corrected
CI	Confidence Interval
DNA	Deoxyribonucleic acid
DTM	Digital Terrain Model
DWD	German Meteorological Service (German: Deutscher Wetterdienst)
FCV	Feline calicivirus
FCoV	Feline coronavirus
FeLV	Feline leukemia virus
FHV-1	Feline herpesvirus 1
FIV	Feline immunodeficiency virus
FPV	Feline parvovirus
GLMMs	Generalised Linear Mixed Effects Models
glmm TMB	Generalized Linear Mixed Models using Template Model Builder
KDE	Kernel Density Estimation
*k*-nn	*k*-nearest neighbors
MEMs	Moran’s Eigenvector Maps
MuMIn	Multi-Model Inference
RNA	Ribonucleic acid
*R*^2^	Coefficient of determination
s.e.	standard error
SRTM	Shuttle Radar Topography Mission
VIFs	Variation Inflation Factors
