# Supplementary material for: Exposure to Feline Viruses in European Wildcats (Felis s. silvestris) in Germany: Spatial Patterns and Environmental Risk Factors
Source: Viruses. 2026 May 29;18(6):627. doi: 10.3390/v18060627 (PMC13307804; doi:10.3390/v18060627)
Supplement: Supplementary file 1 [file viruses-18-00627-s001.zip › viruses-4319858-supplementary.pdf]

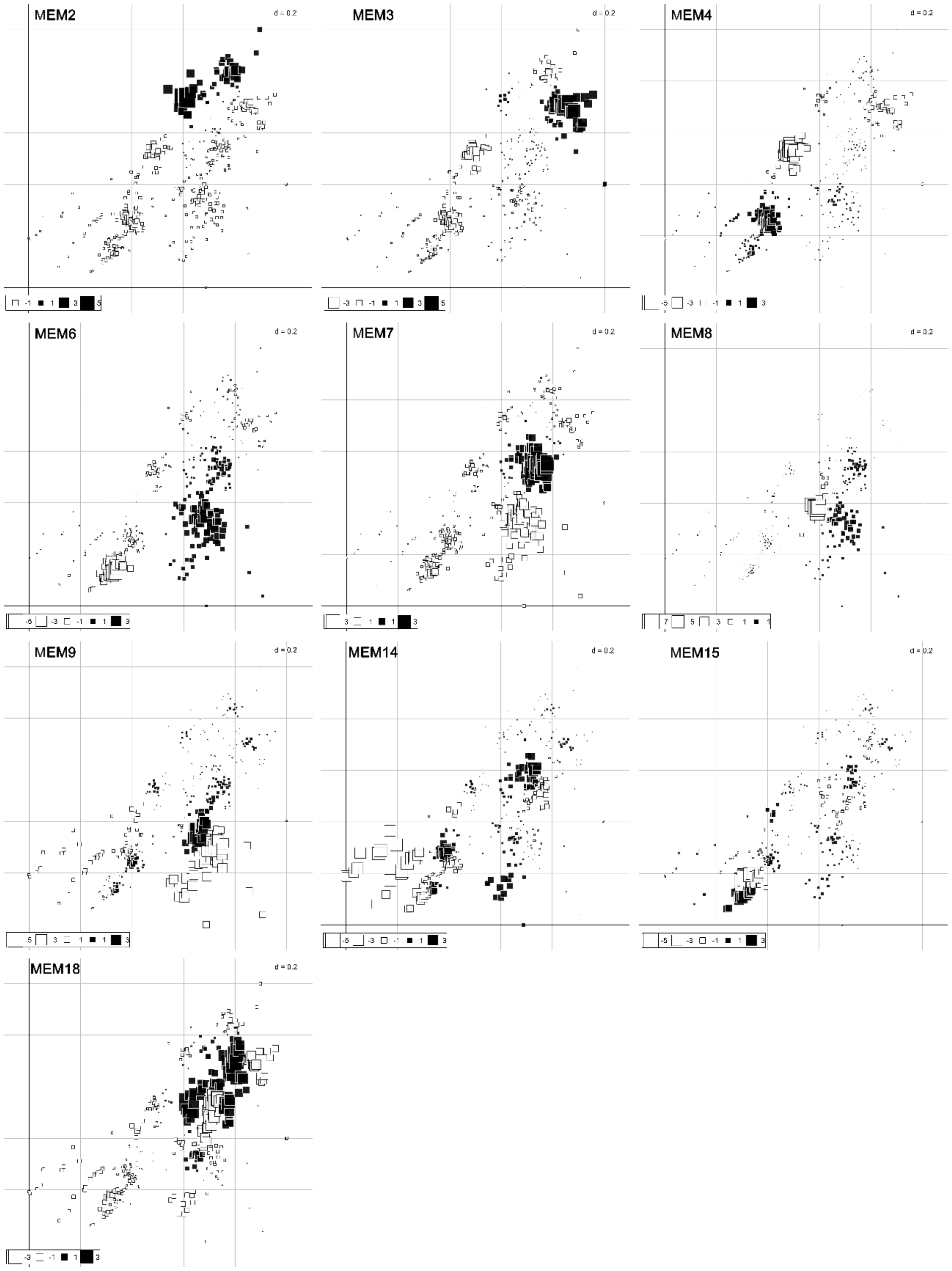

Figure S1: Moran's eigenvector maps (MEM) selected as significant in explaining the spatial autocorrelation of the presence of antigens to feline leukemia virus (FeLV) with a generalized linear mixed model (GLMM) that included only MEMs as fixed factors (see Materials and Methods). Each point represents a sampling site and the size and shading of the symbols corresponds to the magnitude and sign of the eigenvector values, respectively. Axes represent the geographic coordinates (easting and northing) of the sampling locations that have been normalized to range between 0 and 1 using a min-max normalization.

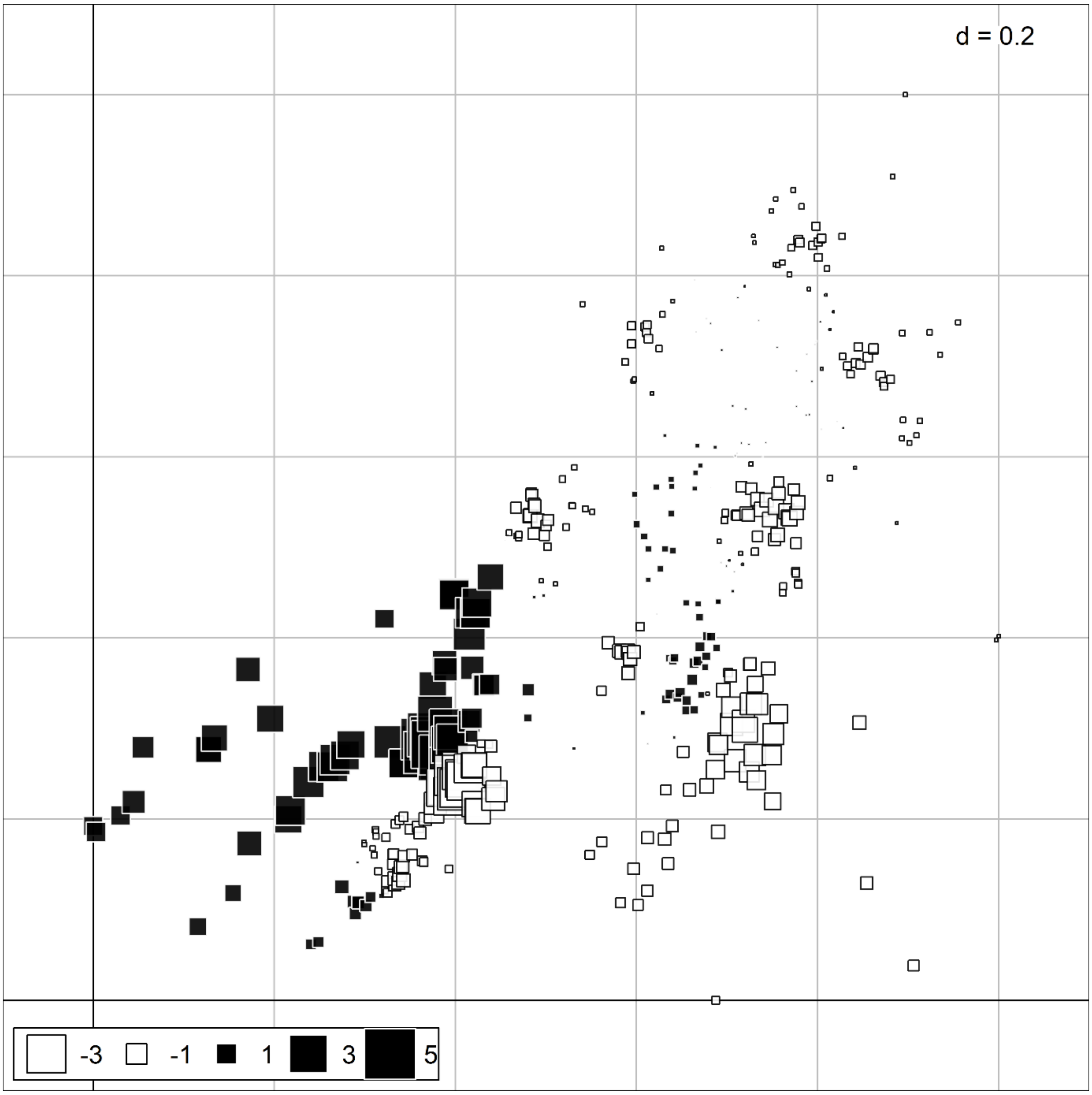

Figure S2: Moran's eigenvector map 10 (MEM10) selected as significant in explaining the spatial autocorrelation of the presence of antibodies to feline parvovirus (FPV) with a generalized linear mixed model (GLMM) that included only MEMs as fixed factors (see Materials and Methods). Each point represents a sampling site and the size and shading of the symbols corresponds to the magnitude and sign of the eigenvector values, respectively. Axes represent the geographic coordinates (easting and northing) of the sampling locations that have been normalized to range between 0 and 1 using a min-max normalization.

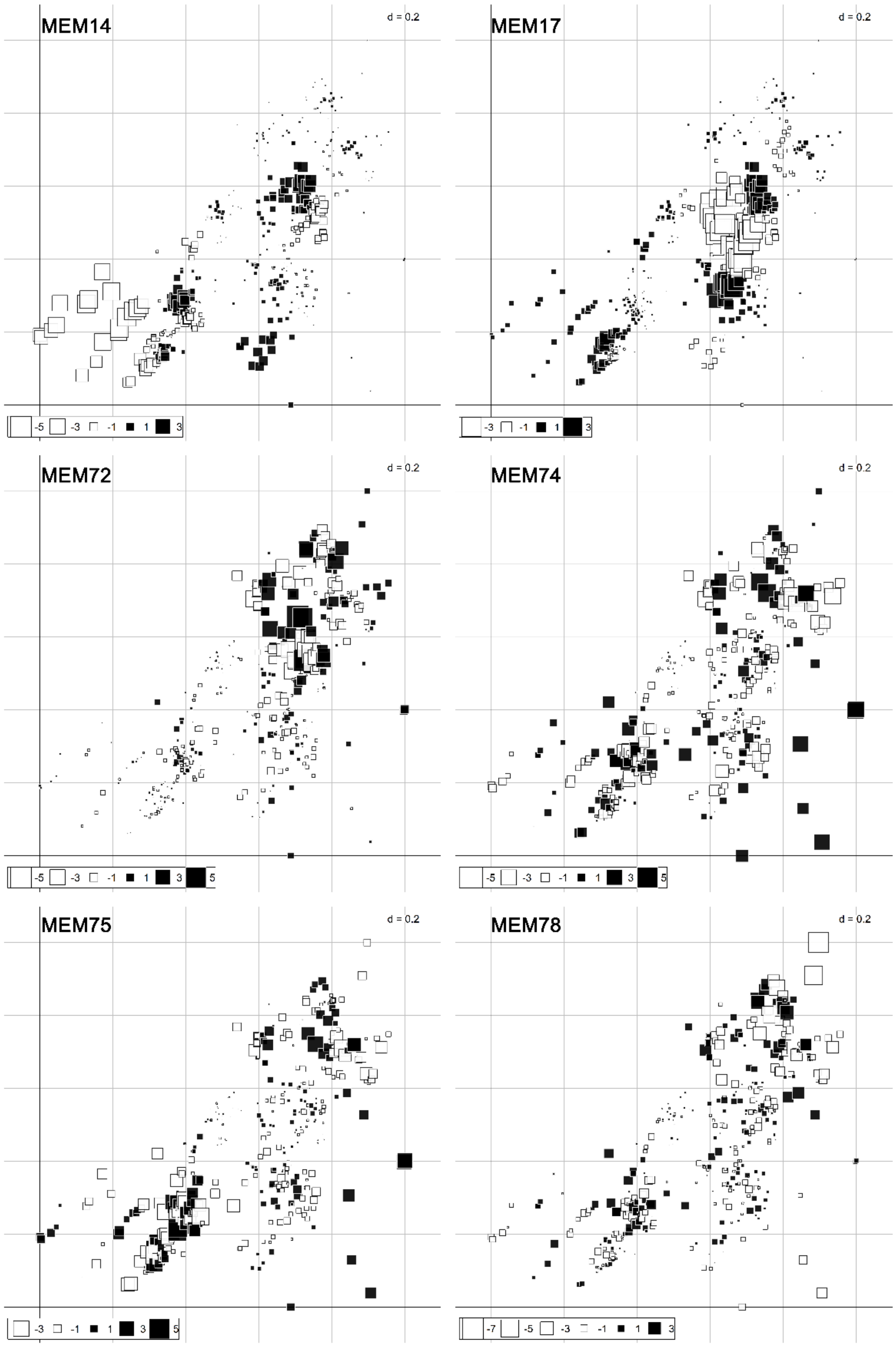

Figure S3: Moran's eigenvector maps (MEMs) selected as significant in explaining the spatial autocorrelation of the presence of antibodies to feline coronavirus (FCoV) with a generalized linear mixed model (GLMM) that included only MEMs as fixed factors (see Materials and Methods). Each point represents a sampling site and the size and shading of the symbols corresponds to the magnitude and sign of the eigenvector values, respectively. Axes represent the geographic coordinates (easting and northing) of the sampling locations that have been normalized to range between 0 and 1 using a min-max normalization.

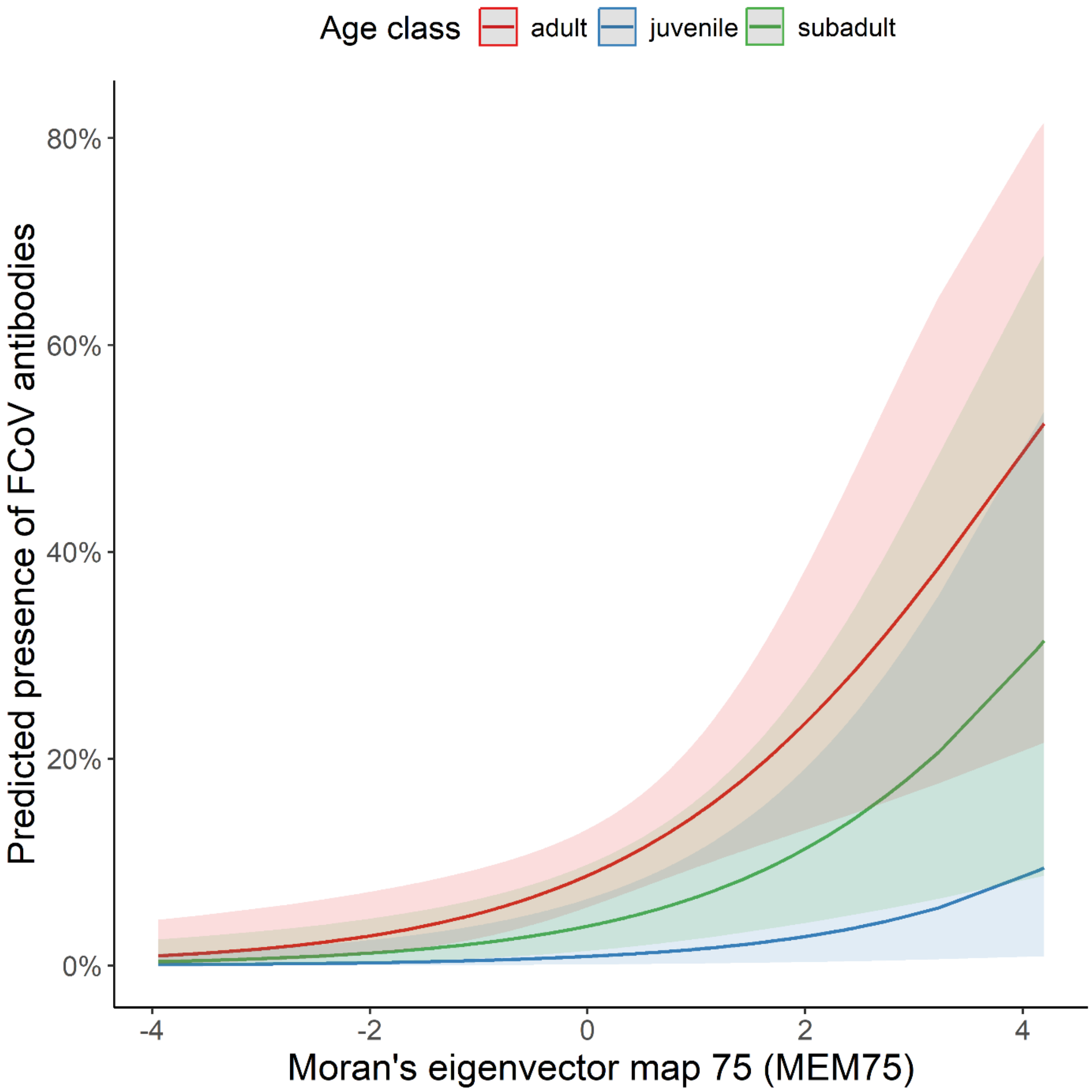

Figure S4: Marginal effects plot of a logistic regression model predicting the presence of feline coronavirus (FCoV) antibodies in German wildcats as a function of (a) age class of the host and the spatial Eigenvector MEM75. The 95% confidence intervals are shown in colour and the plot is based on the most parsimonious model identified after model selection (see Table 3).

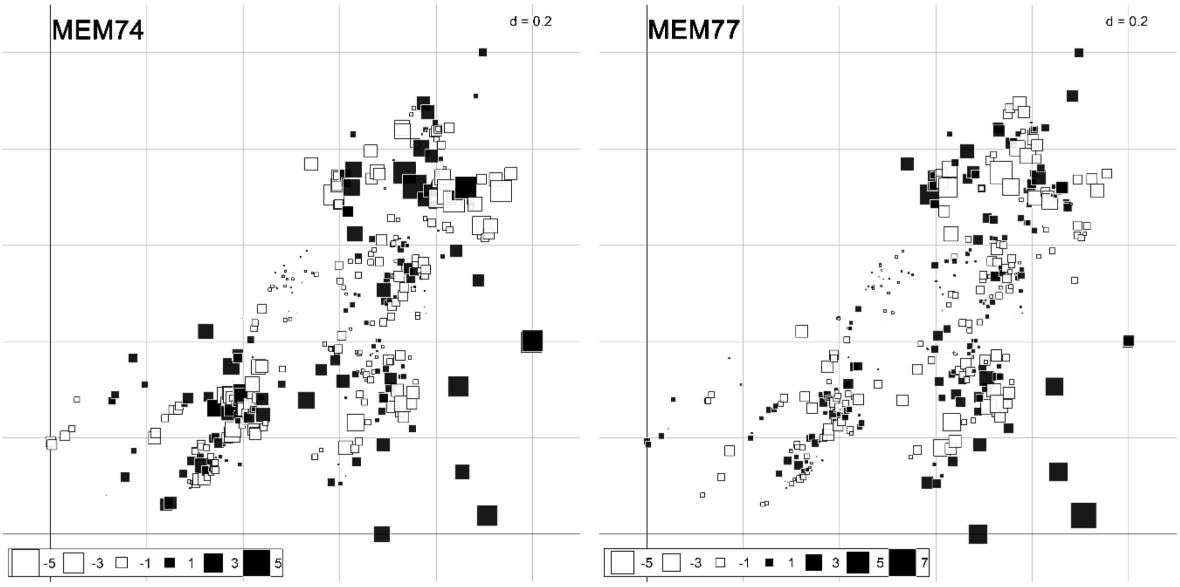

Figure S5: Moran's eigenvector maps (MEMs) selected as significant in explaining the spatial autocorrelation of the presence of antibodies to feline herpesvirus (FHV) with a generalized linear mixed model (GLMM) that included only MEMs as fixed factors (see Materials and Methods). Each point represents a sampling site and the size and shading of the symbols corresponds to the magnitude and sign of the eigenvector values, respectively. Axes represent the geographic coordinates (easting and northing) of the sampling locations that have been normalized to range between 0 and 1 using a min-max normalization.

Table S1: Climatic variables used in our analysis. Data in 1 x 1 km ascii grids obtained from the open data portal (https://opendata.dwd.de/) of the German Meteorological Service (Deutscher Wetterdienst, DWD).

Climatic variable	Index name	Temporal resolution	Explanation
Maximal air temperature	air_temp_max	30 years, for the whole year	Means of the monthly averaged maximum daily air temperature in 2 m height above ground, given in 1/10 °C.
Mean air temperature	air_temp_mean	30 years, for the whole year	Means of the monthly averaged mean daily air temperature in 2 m height above ground, given in 1/10 °C.
Minimal air temperature	air_temp_min	30 years, for the whole year	Means of the monthly averaged minimum daily air temperature in 2 m height above ground, given in 1/10 °C.
Drought index	drought_index	30 years multi-annual	Means of the drought index after de Martonne, unit: mm/°C.
Evapotranspiration-potential	evapo_p	20 years multi-annual	Potential evapotranspiration over grass.
Evapotranspiration-real	evapo_r	20 years multi-annual	Actual evapotranspiration over grass and sandy loam.
Frost days	frost_days	30 years multi-annual	Number of frost days. Definition of frost day: minimum air temperature <0°C.
Hot days	hot_days	30 years multi-annual	Number of days of hot days. Definition of hot day: maximum air temperature >= 30°C.
Ice days	ice_days	30 years multi-annual	Number of days of ice days. Definition of ice day: maximum air temperature < 0°C.
Precipitation	precipitation	30 years, for the whole year	Multi-annual mean of precipitation in mm-
Snowcover days	snowcover_days	30 years multi-annual	Number of days with snow cover. Definition of snow cover: snow depth ≥ 1 cm at morning reading (nowadays 7 UTC).
Summer days	summer_days	30 years multi-annual	Number of summer days. Definition of summer day: maximum air temperature ≥ 25°C.
Sunshine duration	sunshine_duration	30 years, for the whole year	Multi-annual mean of sunshine duration in h.
Begin vegetation period	vegetation_begin	30 years multi-annual	Multi-annual beginning of the vegetation period in Germany in running days of the year (with 28th and 29th Februay counted as a single day).
End vegetation period	vegetation_end	30 years multi-annual	Multi-annual end of the vegetation period in Germany in running days of the year (with 28th and 29th Februay counted as a single day).
Water balance	water_balance	30 years multi-annual	Multi-annual water balance

Table S2: Logistic regressions identifying predictors for the presence of feline leukemia virus (FeLV) antibodies in German wildcats. (a) Presented are results of model averaging based on (a) full averages and (b) conditional averages performed on a subset of best models obtained after performing a model selection procedure on models without interactions. In the initial model, we included latitude, longitude, sex, age class, mean air temperature, precipitation, distance to built-up areas as well as ten Moran's eigenvector maps (MEMs) as fixed factors.

(a)

Coefficients	Estimate	adj. s.e.	*z* value	*p* value
(Intercept)	9.464×10^{1}	2.153×10^{1}	4.396	<0.001
Age class-juvenile	-3.145	7.784×10^{-1}	4.041	<0.001
Age class-subadult	-1.547	3.968×10^{-1}	3.898	<0.001
Latitude	-1.748	4.328×10^{-1}	4.040	<0.001
Longitude	-7.708×10^{-1}	1.932×10^{-1}	3.990	<0.001
MEM18	-4.344×10^{-1}	2.656×10^{-1}	1.635	0.102
Sex-male	3.183×10^{-1}	3.361×10^{-1}	0.947	0.344
MEM8	-3.969×10^{-2}	8.457×10^{-2}	0.469	0.639
MEM3	-4.846×10^{-2}	1.747×10^{-1}	0.277	0.782
MEM2	-4.294×10^{-2}	1.840×10^{-1}	0.233	0.815
Distance to built-up	4.341×10^{-5}	2.892×10^{-4}	0.150	0.881
Mean air t°	-4.323×10^{-4}	5.033×10^{-3}	0.086	0.932
MEM7	-2.921×10^{-3}	4.606×10^{-2}	0.063	0.949
MEM6	1.312×10^{-3}	2.620×10^{-2}	0.050	0.960
Precipitation	1.203×10^{-5}	2.571×10^{-4}	0.047	0.963
Sunshine duration	-4.435×10^{-5}	1.006×10^{-3}	0.044	0.965
MEM14	8.909×10^{-4}	2.448×10^{-2}	0.036	0.971
MEM4	8.464×10^{-4}	2.855×10^{-2}	0.030	0.976
MEM15	2.786×10^{-4}	1.982×10^{-2}	0.014	0.989
MEM9	1.444×10^{-4}	2.445×10^{-2}	0.006	0.995

(b)

Coefficients	Estimate	adj. s.e.	*z* value	*p* value
(Intercept)	9.464×10^{1}	2.153×10^{1}	4.396	<0.001
Age class-juvenile	-3.145	7.784×10^{-1}	4.041	<0.001
Age class-subadult	-1.547	3.968×10^{-1}	3.898	<0.001
Latitude	-1.748	4.328×10^{-1}	4.040	<0.001
Longitude	-7.708×10^{-1}	1.932×10^{-1}	3.990	<0.001
MEM18	-4.681×10^{-1}	2.455×10^{-1}	1.906	0.0566
Sex-male	4.641×10^{-1}	3.115×10^{-1}	1.490	0.1363
MEM8	-1.381×10^{-1}	1.063×10^{-1}	1.300	0.1938
MEM3	-3.024×10^{-1}	3.372×10^{-1}	0.897	0.3697
MEM2	-3.009×10^{-1}	3.996×10^{-1}	0.753	0.4514
Distance to built-up	6.542×10^{-4}	9.277×10^{-4}	0.705	0.4807
Mean air t°	-1.273×10^{-2}	2.428×10^{-2}	0.524	0.6001
MEM7	-9.166×10^{-2}	2.417×10^{-1}	0.379	0.7045
MEM6	4.246×10^{-2}	1.431×10^{-1}	0.297	0.7666
Precipitation	3.908×10^{-4}	1.414×10^{-3}	0.276	0.7823
Sunshine duration	-1.449×10^{-3}	5.567×10^{-3}	0.260	0.7947
MEM14	2.944×10^{-2}	1.377×10^{-1}	0.214	0.8307
MEM4	2.818×10^{-2}	1.624×10^{-1}	0.174	0.8622
MEM15	9.387×10^{-3}	1.147×10^{-1}	0.082	0.9348
MEM9	4.880×10^{-3}	1.421×10^{-1}	0.034	0.9726

Table S3: Logistic regressions identifying predictors for the presence of feline parvovirus (FPV) antibodies in German wildcats. (a) Presented are results of model averaging based on (a) full averages and (b) conditional averages performed on a subset of best models obtained after performing a model selection procedure on models without interactions. In the initial model, we included latitude, longitude, sex, age class, mean air temperature, precipitation, distance to built-up areas as well as one Moran's eigenvector map (MEM10) as fixed factors.

(a)

Coefficients	Estimate	adj. s.e.	*z* value	*p* value
(Intercept)	-5.873×10^{1}	1.793×10^{1}	3.275	0.001
Age class-juvenile	-1.297	4.104×10^{-1}	3.161	0.002
Age class-subadult	-1.313×10^{-1}	2.748×10^{-1}	0.478	0.633
Latitude	8.798×10^{-1}	2.853×10^{-1}	3.084	0.002
MEM10	-6.501×10^{-1}	1.521×10^{-1}	4.274	<0.001
Sunshine duration	8.400×10^{-3}	3.384×10^{-3}	2.482	0.013
precipitation	4.074×10^{-4}	7.431×10^{-4}	0.548	0.584
Longitude	-3.627×10^{-2}	1.040×10^{-1}	0.349	0.727
Sex-male	3.749×10^{-2}	1.307×10^{-1}	0.287	0.774
Distance to built-up	3.664×10^{-5}	2.559×10^{-4}	0.143	0.887
Mean air t°	-7.505×10^{-4}	5.850×10^{-3}	0.128	0.898

(b)

Coefficients	Estimate	adj. s.e.	*z* value	*p* value
(Intercept)	-5.873×10^{1}	1.793×10^{1}	3.275	0.001
Age class-juvenile	-1.297	4.104×10^{-1}	3.161	0.002
Age class-subadult	-1.313×10^{-1}	2.748×10^{-1}	0.478	0.633
Latitude	8.798×10^{-1}	2.853×10^{-1}	3.084	0.002
MEM10	-6.501×10^{-1}	1.521×10^{-1}	4.274	<0.001
Sunshine duration	8.400×10^{-3}	3.384×10^{-3}	2.482	0.013
precipitation	1.104×10^{-3}	8.528×10^{-4}	1.294	0.196
Longitude	-1.604×10^{-1}	1.671×10^{-1}	0.960	0.337
Sex-male	1.917×10^{-1}	2.403×10^{-1}	0.798	0.425
Distance to built-up	4.030×10^{-4}	7.565×10^{-4}	0.533	0.595
Mean air t°	-8.490×10^{-3}	1.793×10^{-2}	0.474	0.636

Table S4: Logistic regressions identifying predictors for the presence of feline coronavirus (FCoV) antibodies in German wildcats. (a) Presented are results of model averaging based on (a) full averages and (b) conditional averages performed on a subset of best models obtained after performing a model selection procedure on models without interactions. In the initial model, we included latitude, longitude, sex, age class, mean air temperature, precipitation, distance to built-up areas as well as six Moran's eigenvector maps (MEMs) as fixed factors.

(a)

Coefficients	Estimate	adj. s.e.	*z* value	*p* value
(Intercept)	-2.785	5.931	0.470	0.639
Age class-juvenile	-2.326	1.043	2.230	0.026
Age class-subadult	-8.018×10^{-1}	5.291×10^{-1}	1.515	0.130
MEM14	2.589×10^{-1}	2.200×10^{-1}	1.177	0.239
MEM17	-4.195×10^{-1}	1.623×10^{-1}	2.585	0.010
MEM72	-4.075×10^{-1}	1.977×10^{-1}	2.061	0.039
MEM74	-4.814×10^{-1}	1.867×10^{-1}	2.578	0.010
MEM75	5.749×10^{-1}	1.848×10^{-1}	3.111	0.002
MEM78	-4.005×10^{-1}	1.790×10^{-1}	2.238	0.023
Sex-male	-3.025×10^{-2}	1.498×10^{-1}	0.202	0.840
precipitation	-7.595×10^{-5}	4.601×10^{-4}	0.165	0.869
Longitude	-8.361×10^{-3}	7.208×10^{-2}	0.116	0.908
Distance to built-up	-4.213×10^{-5}	3.867×10^{-4}	0.109	0.913
Latitude	9.705×10^{-3}	1.081×10^{-1}	0.090	0.928
Sunshine duration	5.621×10^{-5}	1.214×10^{-3}	0.046	0.963
Mean air t°	-1.273×10^{-4}	7.661×10^{-3}	0.017	0.987

(b)

Coefficients	Estimate	adj. s.e.	*z* value	*p* value
(Intercept)	-2.785	5.93	0.470	0.639
Age class-juvenile	-2.326	1.043	2.230	0.026
Age class-subadult	-8.018×10^{-1}	0.529	1.515	0.130
MEM14	3.125×10^{-1}	0.204	1.531	0.126
MEM17	-4.195×10^{-1}	0.162	2.585	0.010
MEM72	-4.075×10^{-1}	0.198	2.061	0.039
MEM74	-4.814×10^{-1}	0.187	2.578	0.010
MEM75	5.749×10^{-1}	0.184	3.111	0.002
MEM78	-4.005×10^{-1}	0.179	2.238	0.025
Sex-male	-2.889×10^{-1}	0.374	0.773	0.440
Precipitation	-8.142×10^{-4}	0.001	0.630	0.528
Longitude	-9.803×10^{-2}	0.228	0.429	0.668
Distance to built-up	-4.987×10^{-4}	0.001	0.402	0.688
Latitude	1.182×10^{-1}	0.360	0.329	0.742
Sunshine duration	7.127×10^{-4}	0.004	0.167	0.867
Mean air t°	-1.634×10^{-3}	0.027	0.060	0.952
